# Identification and Expression Analysis of Sugar Transporter Gene Family in *Aspergillus oryzae*

**DOI:** 10.1155/2020/7146701

**Published:** 2020-11-07

**Authors:** Gongbo Lv, Chunmiao Jiang, Tiantian Liang, Yayi Tu, Xiaojie Cheng, Bin Zeng, Bin He

**Affiliations:** ^1^Jiangxi Key Laboratory of Bioprocess Engineering and Co-Innovation Center for In Vitro Diagnostic Reagents and Devices of Jiangxi Province, College of Life Sciences, Jiangxi Science & Technology Normal University, Nanchang 330013, China; ^2^College of Life Sciences, Sichuan Normal University, Chengdu 610101, China

## Abstract

Sugar transporter (SUT) genes are associated with multiple physiological and biochemical processes in filamentous fungi, such as the response to various stresses. However, limited systematic analysis and functional information of SUT gene family have been available on *Aspergillus oryzae* (*A. oryzae*). To investigate the potential roles of SUTs in *A. oryzae*, we performed an integrative analysis of the SUT gene family in this study. Based on the conserved protein domain search, 127 putative SUT genes were identified in *A. oryzae* and further categorized into eight distinct subfamilies. The result of gene structure and conserved motif analysis illustrated functional similarities among the AoSUT proteins within the same subfamily. Additionally, expression profiles of the AoSUT genes at different growth stages elucidated that most of AoSUT genes have high expression levels at the stationary phase while low in the adaptive phase. Furthermore, expression profiles of AoSUT genes under salt stress showed that AoSUT genes may be closely linked to salt tolerance and involved in sophisticated transcriptional process. The protein-protein interaction network of AoSUT propounded some potentially interacting proteins. A comprehensive overview of the AoSUT gene family will offer new insights into the structural and functional features as well as facilitate further research on the roles of AoSUT genes in response to abiotic stresses.

## 1. Introduction

*Aspergillus oryzae* has been routinely used in the production of traditional fermented food such as soy sauce, miso, vinegar, and fermented soybean paste in Asian countries for centuries [1]. *A. oryzae* has been generally listed as “Generally Recognized as Safe (GRAS)” by the Food and Drug Administration (FDA) in the USA, and its safety was recognized by the World Health Organization (WHO) [2]. *A. oryzae* also has a prominent ability of secreting various hydrolases, liking amylase, protease, and lipase, to degrade large molecules such as polysaccharides and proteins into relatively low-molecular-weight metabolites, including smaller monosaccharides and amino acids [3]. Furthermore, it serves as a source of secondary volatile metabolites that contribute to the quality of fermented products; thus, it is also frequently expected as hosts for heterologous production of useful secondary metabolites [4–7].

Sugars are not only primary nutrients and structural precursors but also signaling molecules that trigger changes in gene regulation for signal transduction and stress responses [8–10]. In fungi, the function of sugar as signaling molecules was mainly achieved through sugar transport and sensing. Considerable researches focused on the transportation and utilization of sugar, such as the absorption and utilization of xylose and glucose [11, 12]. Meanwhile, accumulating researches paid much attention to the ability of stress response of SUT. For example, different SUT families in *Aspergillus niger* (*A. niger*) have a dynamic expression pattern during growth on distinct carbon sources and hence enable the organism to adapt to an extremely broad range of environments [13]. Contrary to *A. niger*, in response to different carbon sources, *Cordyceps militaris* (*C. militaris*) SUT genes within each subfamily harbor an analogous expression pattern with the exception of those involved in the pentose subfamily, which were highly expressed in the xylose culture [14]. Vankuyk et al. reported that regulators CreA regulated the expression of SUT genes in *A. niger* in response to extracellular pH changes [15]. In *Arabidopsis*, overexpression of rice monosaccharide transporter gene (OsMST6) could enhance the tolerance to the salinity [16]. In insects, sugar transporters might also act as receivers for virus entry. It was constitutively expressed in susceptible silkworm, thus leading to the host yield to viral infection [17]. What is more, in response to different stocking densities, intestinal section-dependent expression patterns of the three transporter genes (glut2, sglt1, and sglt4) in grass carp (*Ctenopharyngodon idellus*) did not alter. Nevertheless, high stocking density led to significant differences in segmental mRNA expression levels [18].

Whole transcriptome sequencing gives a valuable alternative to genome-wide sequencing for gene mining and functional identification [19]. It has enabled us to easily gain long-read or full-length transcripts with complete coding sequences (CDS) and characterization of gene families [20]. These approaches have become a primary strategy in characterizing gene expression profiles and elucidating the genetic networks in all fields, such as animals [17, 18], plants [21–23], and fungi [13, 24–26]. However, just a few of the SUTs have been reported in fungi, such as trehalose transporter in *Beauveria bassiana* (BbAGT1), putative pentose transporter in *C. militaris* (CCM_06358), and raffinose transporter in *Metarhizium robertsii* (Mrt) [14, 27, 28]. Nonetheless, there is no systematic analysis and characterization of SUT family genes in *A. oryzae*. Thus, in the present study, we performed the genome-wide identification of evolutionary, gene structures and basic physicochemical properties of AoSUT genes. Furthermore, expression profiles of AoSUT genes at different stages of development stage and in response to salt stress treatment were analyzed using the RNA-Seq data, in an attempt to understand their possible roles in salt stress. The findings of the current investigations will serve as a reference of sugar regulation mechanism for other related *Aspergillus*.

## 2. Materials and Methods

### 2.1. Identification of Sugar Transporter Genes and Physicochemical Properties

The genomic, CDS, and protein sequences of *A. oryzae RIB40* were downloaded from the *Aspergillus* Genome Database (AspGD, http://www.aspergillusgenome.org/). The sugar transporter protein sequences of *Saccharomyces cerevisiae* (ScSUT) were retrieved from the *Saccharomyces* Genome Database (SGD, https://www.yeastgenome.org/). ScSUT proteins were employed as query sequences in a Base Local Alignment Search Tool (BLAST) [29]. To ensure the selected sequences were nonredundant sequences and the presence of the Sugar_tr domain (PF00083) in each candidate AoSUT protein, we downloaded the Sugar_tr domain from the Pfam database [30] (http://pfam.sanger.ac.uk/search). It was used to search the *A. oryzae RIB40* protein database by HMMER software with a standard *E* value < 1 × 10^−5^ [31]. The Sequence Manipulation Suite online tool (http://www.detaibio.com/sms2/protein_iep.html) was used to estimate the basic physicochemical properties of the protein, such as the isoelectric point and molecular weight of the gene product for each member of *A. oryzae* sugar transporter gene family. Thereafter, the subcellular localization of sugar transporter genes was predicted by two online analysis tools, such as WoLF PSORT Prediction (https://wolfpsort.hgc.jp) and LocTree3 Prediction system (https://rostlab.org/services/loctree3/). The results were confirmed by more than one method.

### 2.2. Multiple Sequence Alignment and Phylogenetic Analysis

Multiple sequence alignments and phylogenetic analysis of sugar transporter proteins in *A. oryzae* were subjected to MEGA X [32]. Alignments were performed using the Muscle program with default parameters, and the results after that were subjected to construct unrooted phylogenetic tree with maximum likelihood (ML) using pairwise deletion option and Poisson correction model. To further verify the reliability phylogenetic tree, the Neighbor-Joining (NJ) tree was constructed with the Jones–Taylor–Thornton (JTT) model. Both of the phylogenetic trees were bootstrap value set as 1000 replications in order to choose the best result. The unrooted tree was visualized by the online tool Interactive Tree Of Life (https://itol.embl.de/).

### 2.3. Analysis of Conserved Motifs and Gene Structures

To obtain insights into the diversity of motif compositions in AoSUT proteins, SUT protein sequences were subjected to the MEME (version 5.1.1, http://meme-suite.org/tools/meme) [33]. It is based on the maximum expectation (EM) algorithm, which alternates the execution of steps E (expectation) and M (maximum). Meanwhile, motif sequences and annotations were further predicted by Pfam. MEME using the default parameters with the exception that maximum number of motifs set to 6 and optimum width was from 6 to 50. Following that, the results are visualized by the TBtools software.

To apprehend better the structure of each SUT gene, CDSs and corresponding genomic sequences of AoSUT were downloaded from the *Aspergillus* Genome Database. Gene Structure Display Server 2.0 (GSDS 2.0, http://gsds.cbi.pku.edu.cn/) was used to generate a schematic diagram of exon-intron organization by comparison of the genomic and CDS sequences of each AoSUT gene.

### 2.4. Analysis of Chromosomal Locations

To determine the chromosomal locations of all SUT genes in *A. oryzae*, the information of locus coordinates was obtained from the genomic sequences. The MapChart software was used for the mapping of AoSUT genes' chromosomal positions and relative distances on the basis of their ascending order of physical position (bp).

### 2.5. Expression Analysis of AoSUT Genes in Different Growth Stages and Salt Stress Treatments

The genome-wide transcriptome data of *A. oryzae* in different growth stages and salt stress treatments were obtained from NCBI SRA databases under BioProject Accession PRJNA407002 and PRJNA383095. The raw reads that contained adapter sequence or comprised of more than 10% unknown nucleotide base calls (N) were removed. Gene expression levels were normalized using the FPKM (Fragments Per Kilobase of transcript per Million mapped reads) method. According to the GeneID of AoSUT genes in *A. oryzae* expressed transcripts, the expression data of these genes in three different stages (24, 48, and 72 h, corresponding to the adaptive phase, logarithmic phase, and stationary phase) and four salt stress treatments (cultivated in potato dextrose agar medium supplied with 0, 5, 10, and 15% NaCl) were obtained. The expression profiles were displayed in a heat map generated with the heat map Illustrator software (v 1.0.3.7) by the default data normalization parameter and clustering method [34].

To further ascertain the expression profiles of SUT genes in *A. oryzae*, quantitative real-time RT-PCR (qRT-PCR) experiments of six selected genes (including AoSUT1, 5, 12, 23, 35, and 69) were performed. The genome-wide transcriptome data of *A. oryzae* at three growth stages and four salt stress treatments were obtained from databases. Specifically, three growth stages represent the adaptive, logarithmic, and stationary phases, separately. Four salt stress treatments are equivalent to control, slight stress, moderate stress, and severe stress, respectively. All the transcriptome data are the average of three biological replicates. Total RNA of all collected samples was extracted using the TRIzol Reagent (Takara, Beijing, China) following the instructions in our previous studies [35]. CFX96 Real-Time PCR Detection System (Bio-Rad, CA, USA) was used to perform the qRT-PCR analysis. The specific primers of sugar transporter genes in *A. oryzae* used for qRT-PCR were listed in Table S1. The reaction conditions were 30 s at 94°C, 45 cycles of 20 s at 94°C 20 s at 55°C, and 30 s at 72°C. The melting curves were analyzed from 60°C to 95°C to observe the specificity of the PCR products. The comparative 2^−*ΔΔ*CT^ method was employed to calculate the relative expression between samples [36].

### 2.6. Analysis of the Protein-Protein Interaction Network

Protein-protein interaction (PPI) data were gained from the online database of *STRING* (https://string-db.org/), which is an open source software interface for the exploration, analysis, and visualization of protein-protein associations. These interactions are obtained from text mining of literature of experimental validation including physical interactions and enzymatic reactions found in signal transduction pathways, high-throughput experimental data, and computational predictions containing those based on genomic context analysis and also derived from analyses of coexpressed genes [37]. The PPI data were preprocessed including removing redundancy and self-loops. Targets with a high confidence score > 0.7 were selected to construct the PPI networks. The PPI networks were visualized in Gephic software with the nodes representing proteins/genes and the edges representing interactions between any two proteins/genes.

### 2.7. Statistical Analysis

All values obtained from the studies were presented as mean ± SE. Data from the same period were evaluated by one-way nested analysis of variance (ANOVA), followed by least significant difference test (LSD) for mean comparison. All statistical analysis was performed with SAS 9.20 software (SAS Institute Inc., Cary, North Carolina, USA). Values with *p* < 0.05 were considered significant.

## 3. Results and Discussion

### 3.1. Genome-Wide Identification of SUT Gene Family in *A. oryzae* RIB40

Through a BLAST search and HMMER analysis, eventually, a total of 127 putative SUT genes in the *A. oryzae* RIB40 genome were identified and named based on their chromosomal locations (Table 1). The length of 127 AoSUT proteins varied from 115 (AoSUT102) to 767 (AoSUT69) amino acid residues, and the number of transmembrane domains ranged from 2 (AoSUT102, AoSUT117) to 12 (AoSUT3, AoSUT14, AoSUT15, etc.).

The basic physicochemical properties of 127 AoSUT were estimated by the Sequence Manipulation Suite online tool (Table 1). From the results obtained, the molecular weight of these AoSUT proteins ranged from 13.21 (AoSUT102) to 84.61 (AoSUT69) kDa. What is more, half of the AoSUT proteins exhibited alkaline isoelectric points greater than 7.41, with the highest being 9.61 (AoSUT45), while 25 proteins had acidic isoelectric points of less than 6.5, of which AoSUT50 was the lowest at 4.87. Also, a few proteins, such as AoSUT26, AoSUT38, and AoSUT64, had relatively neutral isoelectric points that fell between 6.5 and 7.41.

WoLF PSORT Prediction and LocTree3 Prediction system were performed to precisely predict the subcellular localization for the products of the AoSUT gene family. Based on the predicted results, overwhelming majority of AoSUT proteins were located in the plasma membrane, demonstrating that these proteins as transcription factors play a transcriptional regulatory role directly in the plasma membrane.

### 3.2. Classification and Phylogenetic Analysis of AoSUT Gene Families

**Figure 1:**
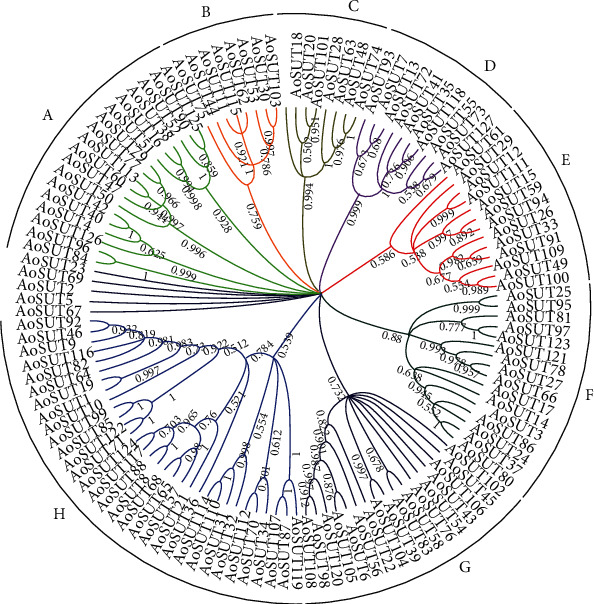
The phylogenetic relationship in *A. oryzae* SUT proteins. The maximum likelihood tree was constructed by the MEGA X with 1000 bootstrap replications. Eight clades were distinguished in different colors, and several unclassified genes were colored in black.

In order to better understand the genetic relationship within members of the SUT gene family in *A. oryzae*, the amino acid sequences of 127 AoSUT were used to construct a maximum likelihood phylogenetic tree. As shown in Figure 1, the AoSUT genes (except AoSUT2, AoSUT5, AoSUT67, AoSUT23, and AoSUT69) were classified into eight different clades according to the research of de Vries et al. [10], which include clades A-H. Additionally, the Neighbor-Joining (NJ) tree of SUT genes with 1000 repeat values displayed a topology which was analogous to ML topology (Figure S1). There were 32 members in the clade H subfamily, which was the largest subfamily, while only 8 members grouped in clades B and C.

These AoSUT genes were grouped together suggesting that these homologous genes may have derived from multiple duplications after the speciation of *A. oryzae* during the evolution.

### 3.3. Conserved Motifs and Structures of AoSUT Gene Families

**Figure 2:**
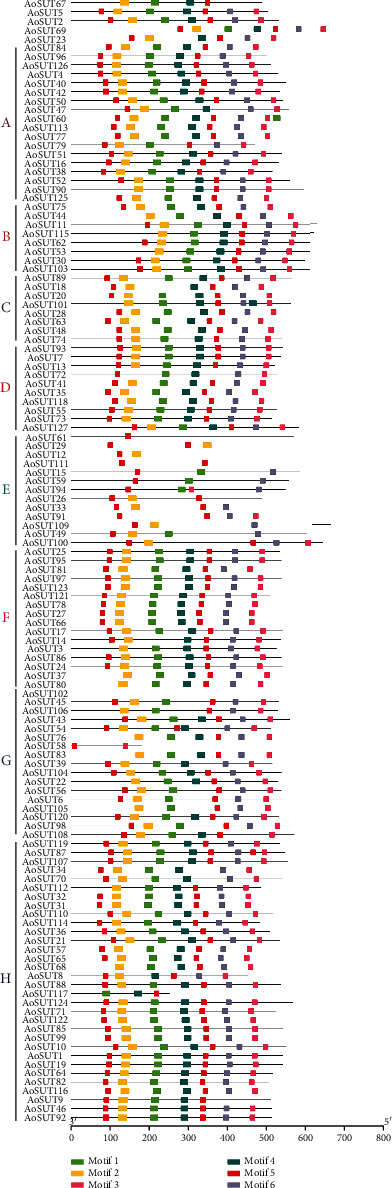
Conserved motifs of the SUT gene family in *A. oryzae*. All conserved motifs of the AoSUT proteins were identified by the MEME program. Protein sequences are indicated by thick gray lines, and the conserved motifs are represented by different colored boxes. The length (the number of amino acids) of the protein and motif can be estimated using the scale bar at the bottom.

A total of six conserved motifs were identified in the SUT gene family of *A. oryzae*, and the location of these motifs within the protein is represented in Figure 2. As the figure shows, motif numbers varied from zero to eight. Motif 5 was included in all of AoSUT (except AoSUT102), and the majority of AoSUT genes contained two motif 5, such as AoSUT27 and AoSUT66. These results elucidated that motif 5 may play a vital role in AoSUT genes. Moreover, only a few protein sequences do not contain motif 2, demonstrating its significance for the AoSUT proteins in *A. oryzae*. And the motif number of several genes is under 3, AoSUT 26, AoSUT 29 and AoSUT 117 (3 motif), AoSUT111 (double motif 5). It is noteworthy that the AoSUT102 have no motif. The consensus amino acid sequence width of the conserved motifs ranged from 15 to 24 amino acids (Table 2). Furthermore, the amino acid frequency in the six conserved motifs of AoSUT proteins was investigated. It showed that the amino acid frequency of the conserved motifs is not consistent in different AoSUT proteins (Figure S2).

To obtain a further insight into the evolutionary trajectory and structural diversity of SUT genes, exon-intron boundaries were analyzed by the GSDS software. As the result showed in Figure S3, a considerable proportion of AoSUT genes contained short and abundant introns, and an analogous phenomenon has been found in the genomes of many lower eukaryotes [38]. The number of exons in AoSUT gene is diversified, ranging from one to 13, whereas the number of introns ranged from zero to 12. It is known that genes lacking introns (e.g., AoSUT99 and AoSUT117) would evolve faster than the rate of intron gain after gene duplication. Simultaneously, it should be noted that some members shared similar intron numbers but with different intron lengths, such as AoSUT19 and AoSUT30 and AoSUT65 and AoSUT69. The existence of plentiful exons and introns indicates that abundant energy is spent to synthesize introns and to maintain the nucleoprotein complex serving the intron-containing genes and that several mechanisms act to sustain vast genomic information [39, 40]. Variation in architectural features of AoSUT genes indicated that gains or losses of exons have occurred during the evolutionary process of the SUT gene family, which may give rise to functional diversity of closely associated SUT genes.

### 3.4. Chromosomal Distribution of AoSUT Genes

**Figure 3:**
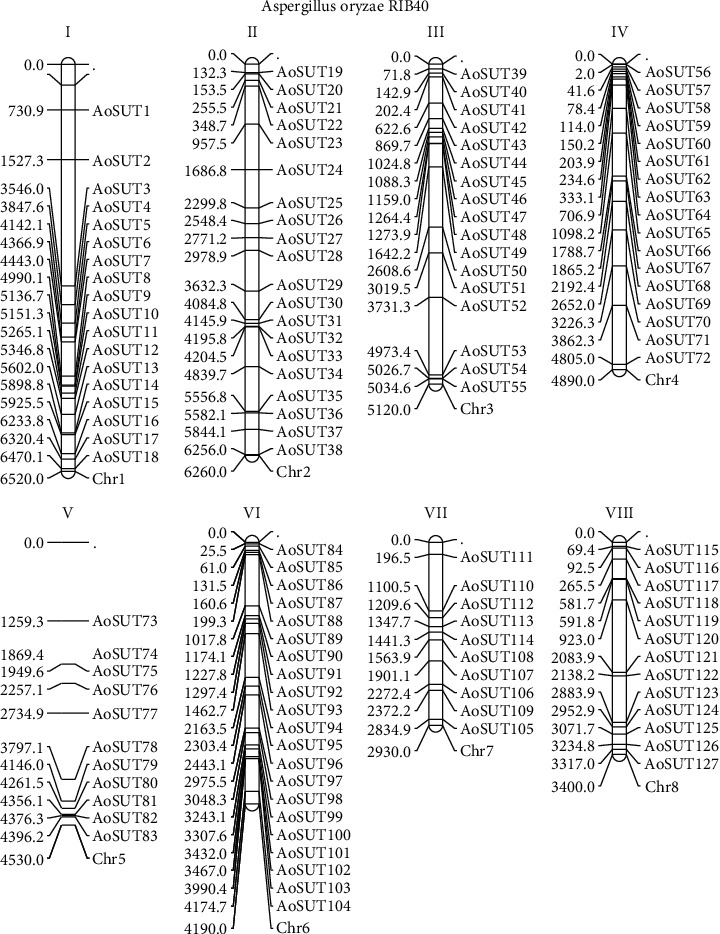
The chromosomal locations of AoSUT genes in the *A. oryzae* genome. Chromosomes I-VIII are depicted as bars. The AoSUT genes are indicated by black lines.

The distribution of 127 SUT genes in *A. oryzae* on 8 chromosomes was visualized by the MapChart program. It can be seen that all of the 127 AoSUT genes are nonrepetitive and randomly distributed. The great amounts of AoSUT genes were located on the proximate or the distal ends of the chromosomes (Figure 3). There were 11 and 10 AoSUT genes mapped onto chromosomes 5 and 7 whereas chromosomes 2 and 6 harbored the most AoSUT genes (20 and 21, respectively). In addition, 17 AoSUT genes were found on chromosomes 3 and 4.

### 3.5. Expression Analysis of AoSUT Genes during Growth Stages

**Figure 4:**
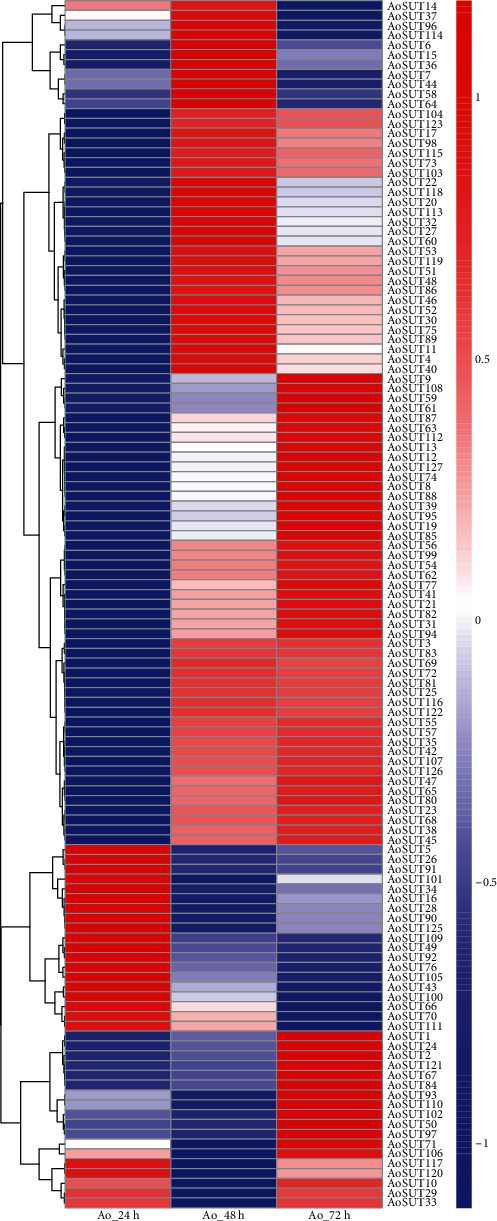
Expression profiles of AoSUT genes at different stages of development. Ao_24_48_72h indicated the expression level of AoSUT at 24 h, 48 h, and 72 h (corresponding to lag phase, logarithmic phase, and stationary phase, respectively). The color of the scale bar, ranging from blue to red, represents low and high expressions, respectively.

To investigate the transcript pattern of SUT family genes during growth stages, the expression patterns at three developmental stages of *A. oryzae* (from adaptive, logarithmic, to stationary phase) were analyzed by mining publicly accessible *A. oryzae* transcriptome datasets, which were released by our previous studies [36]. The results revealed that a large proportion of AoSUT genes are highly expressed at the logarithmic and stationary phases (Figure 4). Moreover, a portion of AoSUT genes showed apparent upregulation in the logarithmic phase, whereas it had low expression level in the stationary phase (e.g., AoSUT7, AoSUT44, and AoSUT96). Similarly, some AoSUT genes have high expression in the stationary phase while low in the adaptive phase (e.g., AoSUT8, AoSUT12, and AoSUT88), revealing that these AoSUT genes may be peculiarly involved in the growing processes during adaptive and logarithmic phases. The diversity of expression profiles of AoSUT genes indicated a broad array of biological functions during the growth and development of *A. oryzae* under normal conditions.

**Figure 5:**
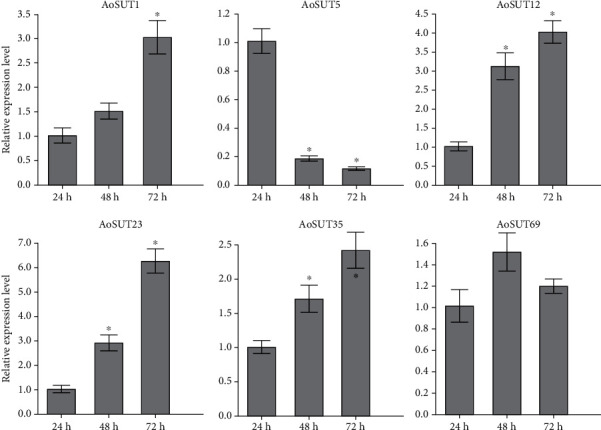
Expression profiles of six selected AoSUT genes at different stages of development determined by qRT-PCR. The bars represent the average (±SE) of biological repeats. Asterisks indicate statistical difference between groups (Student's *t* test): ^∗^*p* < 0.05.

To test the accuracy of the gene expression determined from transcriptome data, six AoSUT genes were randomly selected to investigate the expression profiles at three developmental stages of *A. oryzae* (24 h, 48 h, and 72 h, corresponding to adaptive, logarithmic, and stationary phases) using qRT-PCR; among them the relative expression level of each gene at 24 h is used as a control and its value set to 1 for comparison. As shown in Figure 5, the expression level of AoSUT1, AoSUT12, AoSUT23, and AoSUT35 showed a gradual increase upon transition from the adaptive phase to the logarithmic and stationary phases. However, the expression profiles of AoSUT5 were dramatically decreased from the adaptive phase to the logarithmic and stationary phases. Additionally, the transcript levels of AoSUT69 reached the highest level at the logarithmic phase. The results of qRT-PCR were coordinated with those of transcriptome analysis.

### 3.6. Expression Analysis of AoSUT Genes under Salt Stress

**Figure 6:**
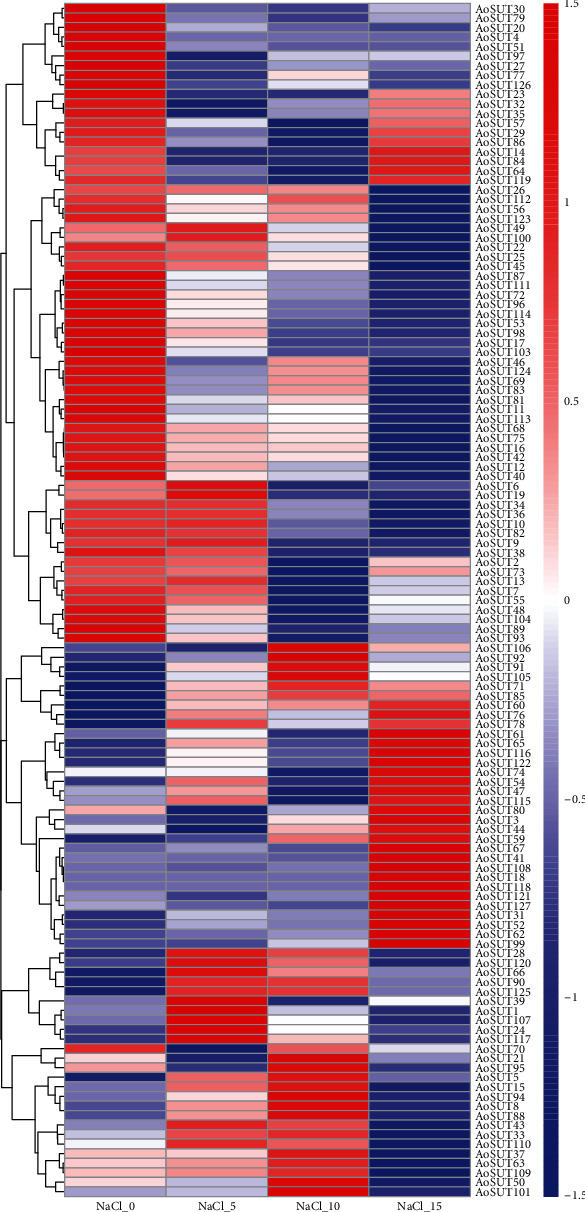
The expression levels of AoSUT genes under salt stress. NaCl_0_5_10_15 indicated samples cultivated in PDA medium supplied with 0, 5, 10, and 15% NaCl, respectively. The color scale bar, ranging from blue to red, represents low and high expressions, separately.

The abiotic stress response ability of SUT genes has been reported in many fungi but not in *A. oryzae*, such as salt stress and ethanol stress. Therefore, we investigated the expression patterns of the AoSUT gene family under different concentrations of salt stress (cultivated in PDA medium supplied with 0, 5, 10, and 15% NaCl) in the present study. The heat map representation of expression patterns of these AoSUT genes, represented by FPKM values, exhibited differential expression patterns (Figure 6). Among them, AoSUT genes presented altered expression patterns, either induction or suppression. A majority of AoSUT genes exhibited relatively high expression in the control, whereas in response to slight and moderate salt stress, a large array of AoSUT genes are not expressed or only expressed at very low levels. There are also a small number of genes (approximately 1/5) that are highly expressed at high concentrations of salt stress. It elucidated that these genes may correlate with responding to salt stress or probably collaborating with other genes associated with the response of salinity. These results obtained indicated that AoSUT are components of a complex transcriptional network regarding the different concentrations of salt stress. The dynamic expression pattern under salt stress illustrated that the mechanism of AoSUT genes in response to osmotic regulation might be sophisticated and varied.

**Figure 7:**
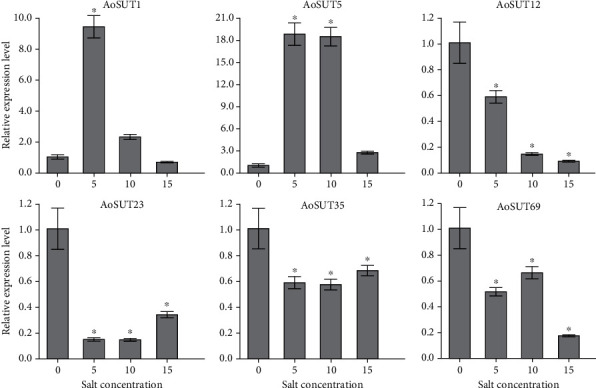
Expression profiles of six selected AoSUT genes in response to salt stress determined by qRT-PCR. The bars represent the average (±SE) of biological repeats. Asterisks indicate statistical difference between groups (Student's *t* test): ^∗^*p* < 0.05.

We also selected six AoSUT genes (including AoSUT1, AoSUT5, AoSUT12, AoSUT23, AoSUT35, and AoSUT69) to examine their expression levels under salt stress using qRT-PCR; of them the relative expression level of each gene at under 0% salt concentration was used as a control and its value set to 1 for comparison. Under salt stress, AoSUT1 was notably upregulated at 5% salinity treatment, around nine times the expression level of control (0% salinity treatment), while AoSUT5 had significantly increased expressions when treated in 5 and 10% salinity (Figure 7). In addition, the other four AoSUT genes (AoSUT12, AoSUT23, AoSUT35, and AoSUT69) were downregulated under salt stress, indicating their negative roles in response to salt stress.

### 3.7. Analysis of AoSUT Protein Function Link Network

**Figure 8:**
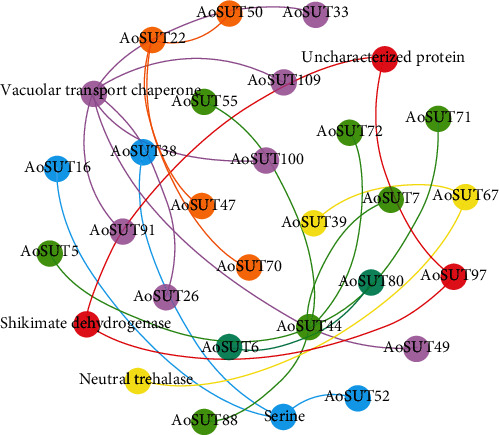
A protein-protein interaction (PPI) network of *A. oryzae* sugar transporter. The nodes represent proteins, and the edges represent the corresponding PPI. The confidence score was required to be greater than 0.7.

To elucidate the potential molecular mechanisms of *A. oryzae* sugar transporter protein, here, we constructed the PPI network using the *STRING* database. Twenty-five AoSUT proteins were included in this network (Figure 8). Besides AoSUT proteins, there were five proteins, including vacuolar transport chaperone, shikimate dehydrogenase, serine, neutral trehalase, and an uncharacterized protein, located in the important positions of the network. Moreover, various types of protein interactions exist in this figure, for instance, AoSUT22 and Serine associated with three proteins, respectively, and AoSUT80 only interacted with AoSUT6. Interestingly, we also found that shikimate dehydrogenase, AoSUT97, and uncharacterized protein interacted with each other. As the whole network shows, AoSUT44 and vacuolar transport chaperone featured prominently in the protein-protein network, illustrating that both of them play essential roles in maintaining the whole protein interactions in this network. The results obtained in the present study will further accelerate the identification of more vital proteins and biological modules which interacted with AoSUT proteins. The detailed information of the proteins in this PPI network is listed in Table S2.

## 4. Discussion

According to literature mining, with considerable diversity in the numbers and types of SUT gene among fungi, insects, and plants, it can be seen that SUT genes are widespread in nature. The accessibility of the *A. oryzae* genome sequence has supplied a great opportunity to identify gene family members. In the current study, we identified a total of 127 AoSUT genes in eight clades (Figure 1). By searching the conserved SUT domain, researcher ascertained 86 putative SUT genes in *A. niger*, and 83 of these genes were classified into nine different clades supported by bootstrap values (over 60%). The remaining SUTs were outside the main clades [13]. What is more, previous studies reported 23 SUT genes in the black truffle *Tuber melanosporum*, which fell into two subgroups of genes: 16 “true” and 7 “potential” sugar transporter genes [41]. We also performed phylogenetic analysis between *A. oryzae*, *A. niger*, and *Tuber melanosporum* SUT proteins (Figure S4). From the result obtained, these SUTs were divided into nine clades and all were in the *A. niger* phylogenetic tree. Moreover, the topology of this phylogenetic tree was analogous to the *A. oryzae* unrooted tree. Among them, the abovementioned several unclassified AoSUT genes were grouped with *A. niger* and *Tuber melanosporum* SUT genes, which indicated the homology with each other. It is worth noting that *Tuber melanosporum* sugar transporter genes primary distributed in clades D-G and clade I. Above, it is not hard to conclude the variations in the distribution and number of SUT genes among filamentous fungi; this difference may be caused by the evolution of different strains in adapting to distinct external environments. Coincidentally, beyond comparative genomics, researchers identified 85 SUTs in *C. militaris*, which is closed to *A. niger*, clustering into nine subfamilies [14]. Moreover, 100 sugar transporter genes in the silkworm *Bombyx mori* genome (categorized into 11 distinct groups in the phylogenetic tree) and 20 SUT genes were identified from the cassava (*Manihot esculenta*) genome, which were further divided into four clades [17, 23]. To sum up, by comparing SUT number and type with other species, our results of the SUT gene family identification together with the previous studies strongly suggest the multiplicity of SUT genes in the biological world. It might be related to biochemical functions of SUT genes in various species. Our effort will be beneficial to explore SUT genes with similar functional characteristics within distinct species in the near future.

Additionally, we noticed that exon and intron numbers of AoSUT gene are various (exons ranging from one to 13, while introns from zero to 12), which showed that the majority of AoSUT genes possess short and abundant introns (Figure S3). The results might also reflect the mechanism of the expansion in genome size of *A. oryzae* to some extent. Comparatively, the exon-intron structure of SUT genes is different from other species. In aphids, for example, a great number of SUT genes have a multiexon structure (with 7-9 exons being typical). Among them, Ap_ST3 harbors 7 exons with alternative splicing giving two different exon 1 sequences in the 5′ untranslated region [42]. In plants, the exon-intron structure of SUT genes is also diversified. For example, the exon-intron structure of SUT genes in strawberry is different in various subfamilies (ranging from one to 40) and has substantial short introns [21], while the exon-intron structure of SUT genes in cabbages is relatively conserved, 72.7% of which were found to have four exons and three introns [22]. The previous studies revealed that the existence of exons and substantial introns played roles in maintaining vast genomic information content [43].

Although numerous studies have reported the crucial roles of SUT genes in sugar transport and responses to biotic and abiotic stresses in many species, such as *A. niger* [13], aphids [42], and wheat [44], the genome-wide expression profile of the SUT gene family was absent in *A. oryzae*. Here, we explored the expression profiles of the AoSUT genes during growth stages and under salt stress. As shown in Figure 4, AoSUT genes are highly expressed at the logarithmic and stationary phases. In these phases, the number of cells increases exponentially, and cells have been proved of vigorous energy and strong adaptability [45, 46]. The expression patterns of AoSUT genes indicate their primary roles in the permeability of cell membrane and stress responses. These results may lead to more directed understanding on the function of AoSUT genes in developmental biology and stress responses. Under salt stress, AoSUT genes presented altered expression patterns, either induction or suppression, indicating that AoSUT are components of a complex transcriptional network regarding the different concentrations of salt stress. The dynamic expression pattern under salt stress illustrated that the mechanism of AoSUT genes in response to osmotic regulation might be sophisticated and diversified. Similar results have been found in wheat. The expression pattern of two sugar transporter genes (SuT4 and SuT5) were differentially regulated during salt stress, which implies that the product of these SUT genes is critical in signal transduction pathway under osmotic stress due to the distinct salt environment [44]. Furthermore, Truernit et al. reported that the expression level of sugar transporter gene (AtSTP4) in *Arabidopsis* was significantly upregulated under pathogen attack and wounding [47]. These results indicate that the SUT genes may play vital roles in response to biotic and abiotic stresses. Despite the appreciable progress in the analysis of sugar transporters in *A. oryzae*, it should be interesting to perform functional genomics experiments, for further clarifying and experimentally demonstrating the important questions concerning the physiological functions of individual AoSUT genes in the near future, for instance, deletion of AoSUT genes in *A. oryzae*.

## 5. Conclusions

In summary, we performed a systematic and comprehensive analysis of the SUT gene family in *A. oryzae*, including phylogenetic relationships, basic classification, conserved motif, gene structure, chromosomal location, and the expression level during growth stages and under salt stress. In addition, qRT-PCR was employed to examine the expression profiles of the AoSUT genes during growth stages and in responses to salt treatments. The results revealed that the AoSUT gene family may mainly participate in the logarithmic and stationary phases of development and be involved in salt stress responses. These results supply an accurate reference of AoSUT genes, which lay a solid foundation for further characterization of the physiological and biochemical functions of individual AoSUT genes.

## Figures and Tables

**Table 1:** The member of SUT gene family in *A. oryzae*.

Nomenclature	AspGeneID	CDS length (bp)	PL (aa)	PI	MW (kDa)	TMD	SCLpred
AoSUT1	ASPL0000299362	1626	541	6.12	60.46	10	Plas
AoSUT2	ASPL0000348493	1596	531	8.24	58.64	10	Plas
AoSUT3	ASPL0000312952	1575	524	8.96	58.05	12	Plas
AoSUT4	ASPL0000330204	1590	529	8.64	58.34	12	Plas
AoSUT5	ASPL0000321372	1512	503	9.01	54.12	12	Plas
AoSUT6	ASPL0000298924	1635	544	7.06	60.5	11	Vacu
AoSUT7	ASPL0000350553	1608	535	8.15	59.69	12	Plas
AoSUT8	ASPL0000291308	1359	452	7.9	50.65	8	Plas
AoSUT9	ASPL0000301060	1533	510	9.28	55.97	10	Plas
AoSUT10	ASPL0000309712	1653	550	5.56	60.88	10	Plas
AoSUT11	ASPL0000344279	1899	632	7.22	71.65	10	Plas
AoSUT12	ASPL0000349681	1527	508	6.84	55.73	11	Plas
AoSUT13	ASPL0000301678	1563	520	7.29	57.38	12	Plas
AoSUT14	ASPL0000315710	1611	536	6.71	59.68	12	Plas
AoSUT15	ASPL0000287472	1755	584	8.59	62.91	12	Plas
AoSUT16	ASPL0000281504	1593	530	8.28	57.28	12	Plas
AoSUT17	ASPL0000337017	1629	542	8.18	59.11	12	Plas
AoSUT18	ASPL0000349981	1623	540	7.89	59.38	12	Plas
AoSUT19	ASPL0000323998	1623	540	7.49	60.5	8	Plas
AoSUT20	ASPL0000284520	1734	577	7.46	62.29	12	Plas
AoSUT21	ASPL0000322602	1602	533	6.66	59.46	9	Plas
AoSUT22	ASPL0000325484	1590	529	7.68	58.88	12	Plas
AoSUT23	ASPL0000294580	1710	569	7.99	63.22	11	Vacu
AoSUT24	ASPL0000288152	1626	541	9.28	59.47	12	Mito
AoSUT25	ASPL0000309860	1605	534	7.63	58.9	12	Plas
AoSUT26	ASPL0000279704	1464	487	7.08	53.42	11	ER
AoSUT27	ASPL0000335285	1485	494	8.91	54.07	11	Plas
AoSUT28	ASPL0000313172	1671	556	4.98	61.52	12	Plas
AoSUT29	ASPL0000283168	1299	432	8.89	46.44	12	Plas
AoSUT30	ASPL0000281742	1797	598	8.44	66.54	12	Plas
AoSUT31	ASPL0000324124	1515	504	7.88	54.98	11	Plas
AoSUT32	ASPL0000327738	1446	481	9.04	53.16	12	Plas
AoSUT33	ASPL0000334740	1506	501	6.51	54.28	11	Plas
AoSUT34	ASPL0000280136	1503	500	6.25	54.06	12	Plas
AoSUT35	ASPL0000300822	1575	524	6.78	58.61	12	Plas
AoSUT36	ASPL0000349699	1527	508	6.58	55.65	10	Plas
AoSUT37	ASPL0000338681	1671	556	8.11	61.07	12	Plas
AoSUT38	ASPL0000283036	1545	514	6.99	56.61	12	Plas
AoSUT39	ASPL0000289632	1545	514	6.36	56.07	10	Plas
AoSUT40	ASPL0000304806	1650	549	6.97	60.71	10	Plas
AoSUT41	ASPL0000340951	1578	525	6.24	57.49	10	Plas
AoSUT42	ASPL0000295608	1602	533	6.5	58.35	10	Plas
AoSUT43	ASPL0000337719	1680	559	6.08	61.71	10	Mito
AoSUT44	ASPL0000282628	1824	607	8.58	68.4	9	Plas
AoSUT45	ASPL0000346097	1593	530	9.61	58.02	12	Plas
AoSUT46	ASPL0000299188	1542	513	7.78	56.59	10	Plas
AoSUT47	ASPL0000294920	1671	556	8.85	60.69	12	Plas
AoSUT48	ASPL0000341497	1644	547	6.11	59.38	12	Plas
AoSUT49	ASPL0000284342	1809	602	9.08	67.21	12	Plas
AoSUT50	ASPL0000322158	1629	542	4.87	58.75	12	Plas
AoSUT51	ASPL0000327028	1617	538	7.03	59.01	12	Plas
AoSUT52	ASPL0000345761	1680	559	6.58	61.86	12	Mito
AoSUT53	ASPL0000288766	1842	613	7.26	67.86	12	Plas
AoSUT54	ASPL0000348009	1536	511	7.46	55.9	10	Plas
AoSUT55	ASPL0000308756	1578	525	7.78	56.95	9	Plas
AoSUT56	ASPL0000288748	1614	537	8.16	59.3	12	Plas
AoSUT57	ASPL0000342947	1521	506	6.74	56.62	12	Plas
AoSUT58	ASPL0000289192	546	181	8.33	19.9	4	Plas
AoSUT59	ASPL0000302542	1674	557	6.82	60.12	11	Pero
AoSUT60	ASPL0000297440	1623	540	6.31	57.65	12	Plas
AoSUT61	ASPL0000301470	1710	569	5.37	63.49	11	Vacu
AoSUT62	ASPL0000317896	1830	609	7.54	70.1	12	Plas
AoSUT63	ASPL0000305684	1587	528	8.63	58.55	12	Plas
AoSUT64	ASPL0000290346	1548	515	7.03	56.35	12	Plas
AoSUT65	ASPL0000290728	1548	515	6.29	56.95	10	Plas
AoSUT66	ASPL0000314400	1476	491	7.77	54.85	12	Plas
AoSUT67	ASPL0000307186	1467	488	9.14	51.94	12	Vacu
AoSUT68	ASPL0000322566	1488	495	8.03	55.42	12	Plas
AoSUT69	ASPL0000284218	2304	767	7.26	84.61	9	Plas
AoSUT70	ASPL0000291632	1617	538	6.76	59.05	12	Vacu
AoSUT71	ASPL0000316294	1572	523	5.71	57.42	12	Plas
AoSUT72	ASPL0000340407	1590	529	8.1	59.65	10	Vacu
AoSUT73	ASPL0000321912	1542	513	6.18	56.75	10	Plas
AoSUT74	ASPL0000327626	1620	539	6.6	58.86	12	Plas
AoSUT75	ASPL0000291430	1653	550	6.28	60.89	11	Plas
AoSUT76	ASPL0000330168	1641	546	8.88	59.22	12	Plas
AoSUT77	ASPL0000299472	1638	545	8.61	58.72	11	Plas
AoSUT78	ASPL0000348593	1566	521	6.5	56.46	12	Plas
AoSUT79	ASPL0000304244	1413	470	6.5	50.61	9	Plas
AoSUT80	ASPL0000322394	1482	493	8.62	53.88	10	Mito
AoSUT81	ASPL0000308688	1581	526	9.36	58.33	9	Plas
AoSUT82	ASPL0000348347	1521	506	7.37	55.82	12	Plas
AoSUT83	ASPL0000296900	1629	542	8.81	59.7	10	Plas
AoSUT84	ASPL0000327800	1593	530	6.7	57.98	12	Plas
AoSUT85	ASPL0000308836	1629	542	5.5	59.45	12	Plas
AoSUT86	ASPL0000284922	1614	537	7.22	59.13	12	Plas
AoSUT87	ASPL0000306294	1641	546	6.01	60.5	9	Plas
AoSUT88	ASPL0000310270	1611	536	6.98	59.53	10	Plas
AoSUT89	ASPL0000299794	1698	565	6.75	61.76	12	Plas
AoSUT90	ASPL0000343545	1788	595	9.47	66.32	12	Mito
AoSUT91	ASPL0000300438	1485	494	8.37	54.34	10	Plas
AoSUT92	ASPL0000336593	1542	513	6.39	56.75	12	Plas
AoSUT93	ASPL0000344697	1626	541	6.27	60.41	11	Vacu
AoSUT94	ASPL0000326072	1644	547	7.41	59.46	12	Plas
AoSUT95	ASPL0000304476	1614	537	8.81	59.73	12	Plas
AoSUT96	ASPL0000298998	1506	501	6.63	56.33	9	Plas
AoSUT97	ASPL0000307814	1614	537	7.49	60.39	11	Plas
AoSUT98	ASPL0000309592	1698	565	7.75	62.88	12	Plas
AoSUT99	ASPL0000281934	1629	542	5.44	59.99	10	Plas
AoSUT100	ASPL0000287394	1938	645	6.63	70.99	12	Plas
AoSUT101	ASPL0000289388	1689	562	6.08	62.31	12	Plas
AoSUT102	ASPL0000342469	348	115	9.15	13.21	2	Mito
AoSUT103	ASPL0000331538	1887	628	7.91	70.76	11	Vacu
AoSUT104	ASPL0000342747	1614	537	8.23	58.96	12	Plas
AoSUT105	ASPL0000300304	1662	553	7.77	62.09	12	Mito
AoSUT106	ASPL0000294524	1587	528	8.3	58.1	12	Plas
AoSUT107	ASPL0000295892	1665	554	6.87	61.72	12	Plas
AoSUT108	ASPL0000335375	1713	570	6.65	63.51	10	Plas
AoSUT109	ASPL0000333426	2004	667	7.33	75.14	9	ER
AoSUT110	ASPL0000313766	1554	517	6.74	57.24	11	Plas
AoSUT111	ASPL0000328104	1539	512	9.42	55.9	10	Vacu
AoSUT112	ASPL0000334670	1461	486	6.52	53.39	12	Plas
AoSUT113	ASPL0000340285	1584	527	6.31	57.33	12	Plas
AoSUT114	ASPL0000324582	1452	483	6.15	53.48	12	Plas
AoSUT115	ASPL0000284234	1872	623	7.23	70.73	8	Plas
AoSUT116	ASPL0000322906	1659	552	6.87	60.77	10	Plas
AoSUT117	ASPL0000323794	759	252	8.69	29.14	2	Plas
AoSUT118	ASPL0000332556	1596	531	8.49	59.42	12	Plas
AoSUT119	ASPL0000315516	1599	532	7.97	58.21	11	Plas
AoSUT120	ASPL0000348251	1596	531	8.45	58.98	9	Plas
AoSUT121	ASPL0000282924	1527	508	6.79	55.12	12	Plas
AoSUT122	ASPL0000342577	1614	537	5.94	59.11	10	Plas
AoSUT123	ASPL0000324822	1620	539	5.54	60.26	11	Plas
AoSUT124	ASPL0000301868	1701	566	9.31	63.52	12	Plas
AoSUT125	ASPL0000316738	1641	546	6.51	60.49	12	Mito
AoSUT126	ASPL0000349221	1536	511	7.85	56.35	12	Mito
AoSUT127	ASPL0000331760	1749	582	8.56	64.2	12	Plas

PL: protein length; MW: molecular weight; pI: isoelectric point; TMD: transmembrane domain number; SCLpred: predicted subcellular localization; Plas: plasma membrane; Vacu: vacuolar membrane; Mito: mitochondrion; ER: endoplasmic reticulum; Pero: peroxisome membrane.

**Table 2:** The motifs identified in AoSUT proteins.

Motif	Conserved amino acid sequences	*E* values	Sites	Width
1	LPESPRWLVRKGRHEEARQVL	7.3*e* − 980	111	21
2	RFIAGJGVGILSATVPVYQSEIAP	1.0*e* − 886	113	24
3	PETKGRSLEEJDELF	6.0*e* − 755	113	15
4	QFFQQLSGINAISYYAPTJFZ	2.4*e* − 661	101	21
5	FLIDRFGRRKLLLIG	1.8*e* − 498	126	15
6	VPWLYPAEIFPLRLRA	3.0*e* − 442	117	16

## Data Availability

Raw transcriptome data are available through the NCBI Sequence Read Archive databases (BioProject Accession PRJNA407002 and PRJNA383095). All samples were sequenced as 100 bp paired-end reads on an Illumina HiSeq 2000 sequencer.

## Supplementary Materials

Supplementary 1 Table S1: primer details for genes selected for qRT-PCR analysis in this study.

Supplementary 2 Table S2: the detailed information of the AoSUT proteins in the PPI network.

Supplementary 3 Figure S1: phylogenetic analysis of *A. oryzae* SUT proteins. An unrooted NJ tree of *A. oryzae* SUT amino acid sequences using 1000 bootstrap replicates by MEGA X. The eight clades are marked by different colors.

Supplementary 4 Figure S2: sequence logos for 6 motifs of sugar transporter (SUT) domains using the MEME program. MEME motifs are displayed by stacks of letters at each site. The total height of the stack is the “information content” of that site in the motif in bits. The height of each letter in a stack is the probability of the letter at that site multiplied by the total information content of the stack. Abscissa represents the width of the motif, and ordinate represents the bits of each letter, separately.

Supplementary 5 Figure S3: exon-intron structure of 127 sugar transporter genes identified in *A. oryzae*. Exons are shown as red boxes, and introns are shown as black lines.

Supplementary 6 Figure S4: the phylogenetic relationship between *A*. *oryzae*, *A*. *niger*, and *Tuber melanosporum* SUT proteins. The maximum likelihood tree was constructed by the MEGA X with 1000 bootstrap replications. Nine clades were distinguished in different colors.

## References

[1] Zhong Y., Lu X., Xing L., Ho S. W. A., Kwan H. S. (2018). Genomic and transcriptomic comparison of Aspergillus oryzae strains: a case study in soy sauce koji fermentation. *Journal of Industrial Microbiology & Biotechnology*.

[2] He B., Hu Z., Ma L. (2018). Transcriptome analysis of different growth stages of Aspergillus oryzae reveals dynamic changes of distinct classes of genes during growth. *BMC Microbiology*.

[3] Smit G., Smit B. A., Engels W. J. (2005). Flavour formation by lactic acid bacteria and biochemical flavour profiling of cheese products. *FEMS Microbiology Reviews*.

[4] Feng Y., Cui C., Zhao H., Gao X., Zhao M., Sun W. (2013). Effect of koji fermentation on generation of volatile compounds in soy sauce production. *International Journal of Food Science & Technology*.

[5] Kum S.-J., Yang S.-O., Lee S. M. (2015). Effects of Aspergillus species inoculation and their enzymatic activities on the formation of volatile components in fermented soybean paste (doenjang). *Journal of Agricultural and Food Chemistry*.

[6] Munawar A., Marshall J. W., Cox R. J., Bailey A. M., Lazarus C. M. (2013). Isolation and characterisation of a ferrirhodin synthetase gene from the sugarcane pathogen Fusarium sacchari. *Chembiochem*.

[7] Fujii R., Minami A., Tsukagoshi T. (2014). Total biosynthesis of diterpene aphidicolin, a specific inhibitor of DNA polymerase alpha: heterologous expression of four biosynthetic genes in Aspergillus oryzae. *Bioscience, Biotechnology, and Biochemistry*.

[8] Walmsley A. R., Barrett M. P., Bringaud F., Gould G. W. (1998). Sugar transporters from bacteria, parasites and mammals: structure–activity relationships. *Trends in Biochemical Sciences*.

[9] Gupta A. K., Kaur N. (2005). Sugar signalling and gene expression in relation to carbohydrate metabolism under abiotic stresses in plants. *Journal of Biosciences*.

[10] de Vries R. P., Riley R., Wiebenga A. (2017). Comparative genomics reveals high biological diversity and specific adaptations in the industrially and medically important fungal genus Aspergillus. *Genome Biology*.

[11] Colabardini A., Ries L. N., Brown N. (2014). Functional characterization of a xylose transporter in Aspergillus nidulans. *Biotechnology for Biofuels*.

[12] dos Reis T. F., Menino J. F., Bom V. L. P. (2013). Identification of glucose transporters in Aspergillus nidulans. *PLoS One*.

[13] Peng M., Aguilar-Pontes M. V., de Vries R. P., Makela M. R. (2018). In silico analysis of putative sugar transporter genes in Aspergillus niger using phylogeny and comparative transcriptomics. *Frontiers in Microbiology*.

[14] Sirithep K., Xiao F., Raethong N. (2020). Probing carbon utilization of Cordyceps militaris by sugar transportome and protein structural analysis. *Cells*.

[15] van Kuyk P. A., Diderich J. A., Mac Cabe A. P., Hererro O., Ruijter G. J. G., Visser J. (2004). Aspergillus niger mstA encodes a high-affinity sugar/H+ is regulated in response to extracellular pH. *Biochemical Society*.

[16] Monfared H. H., Chew J. K., Azizi P. (2020). Overexpression of a rice monosaccharide transporter gene (OsMST6) confers enhanced tolerance to drought and salinity stress in Arabidopsis thaliana. *Plant Molecular Biology Reporter*.

[17] Govindaraj L., Gupta T., Esvaran V. G., Awasthi A. K., Ponnuvel K. M. (2016). Genome-wide identification, characterization of sugar transporter genes in the silkworm Bombyx mori and role in Bombyx mori nucleopolyhedrovirus (BmNPV) infection. *Gene*.

[18] Liang X., Yan F., Gao Y. (2020). Sugar transporter genes in grass carp (Ctenopharyngodon idellus): molecular cloning, characterization, and expression in response to different stocking densities. *Fish Physiology and Biochemistry*.

[19] Wang Z., Gerstein M., Snyder M. (2009). RNA-Seq: a revolutionary tool for transcriptomics. *Nature Reviews. Genetics*.

[20] Koren S., Schatz M. C., Walenz B. P. (2012). Hybrid error correction and de novo assembly of single-molecule sequencing reads. *Nature Biotechnology*.

[21] Jiu S., Haider M. S., Kurjogi M. M., Zhang K., Zhu X., Fang J. (2018). Genome-wide characterization and expression analysis of sugar transporter family genes in woodland strawberry. *Plant Genome*.

[22] Zhang W., Wang S., Yu F. (2019). Genome-wide identification and expression profiling of sugar transporter protein (STP) family genes in cabbage (Brassica oleracea var. capitata L.) reveals their involvement in clubroot disease responses. *Genes*.

[23] Liu Q., Dang H., Chen Z. (2018). Genome-wide identification, expression, and functional analysis of the sugar transporter gene family in cassava (Manihot esculenta). *International Journal of Molecular Sciences*.

[24] Nogueira K. M. V., de Paula R. G., Antoniêto A. C. C. (2018). Characterization of a novel sugar transporter involved in sugarcane bagasse degradation in Trichoderma reesei. *Biotechnology for Biofuels*.

[25] Milner D. S., Attah V., Cook E. (2019). Environment-dependent fitness gains can be driven by horizontal gene transfer of transporter-encoding genes. *Proceedings of the National Academy of Sciences*.

[26] Sloothaak J., Tamayo-Ramos J. A., Odoni D. I. (2016). Identification and functional characterization of novel xylose transporters from the cell factories Aspergillus niger and Trichoderma reesei. *Biotechnology for Biofuels*.

[27] Fang W., Leger R. J. S. (2010). Mrt, a gene unique to fungi, encodes an oligosaccharide transporter and facilitates rhizosphere competency in Metarhizium robertsii. *Plant Physiology*.

[28] Wang X. X., Ji X. P., Li J. X., Keyhani N. O., Feng M. G., Ying S. H. (2013). A putative *α*-glucoside transporter gene BbAGT1 contributes to carbohydrate utilization, growth, conidiation and virulence of filamentous entomopathogenic fungus Beauveria bassiana. *Research in Microbiology*.

[29] Stephen T. L. M., Altschul F., Schäffer A. A. (1997). Gapped BLAST and PSI-BLAST: a new generation of protein database search programs. *Nucleic Acids Research*.

[30] El-Gebali S., Mistry J., Bateman A. (2019). The Pfam protein families database in 2019. *Nucleic Acids Research*.

[31] Finn R. D., Clements J., Eddy S. R. (2011). HMMER web server: interactive sequence similarity searching. *Nucleic Acids Research*.

[32] Kumar S., Stecher G., Li M., Knyaz C., Tamura K. (2018). MEGA X: molecular evolutionary genetics analysis across computing platforms. *Molecular Biology and Evolution*.

[33] Bailey T. L., Boden M., Buske F. A. (2009). MEME SUITE: tools for motif discovery and searching. *Nucleic Acids Research*.

[34] Tang W., Tu Y., Cheng X. (2019). Genome-wide identification and expression profile of the MADS-box gene family in Erigeron breviscapus. *PLoS One*.

[35] Tang W., Ouyang C., Liu L. (2018). Genome-wide identification of the fatty acid desaturases gene family in four Aspergillus species and their expression profile in Aspergillus oryzae. *AMB Express*.

[36] He B., Ma L., Hu Z. (2018). Deep sequencing analysis of transcriptomes in Aspergillus oryzae in response to salinity stress. *Applied Microbiology and Biotechnology*.

[37] Raman K. (2010). Construction and analysis of protein-protein interaction networks. *Automated Experimentation*.

[38] Loftus B. J., Fung E., Roncaglia P. (2005). The genome of the basidiomycetous yeast and human pathogen Cryptococcus neoformans. *Science*.

[39] Ivashchenko A. T., Tauasarova M. I., Atambayeva S. A. (2009). Exon-intron structure of genes in complete fungal genomes. *Molecular Biology*.

[40] Bondarenko V. S., Gelfand M. S. (2016). Evolution of the exon-intron structure in ciliate genomes. *PLoS One*.

[41] Ceccaroli P., Saltarelli R., Polidori E. (2015). Sugar transporters in the black truffle Tuber melanosporum: from gene prediction to functional characterization. *Fungal Genetics and Biology*.

[42] Price D. R. G., Tibbles K., Shigenobu S. (2010). Sugar transporters of the major facilitator superfamily in aphids; from gene prediction to functional characterization. *Insect Molecular Biology*.

[43] Kupfer D. M., Drabenstot S. D., Buchanan K. L. (2004). Introns and splicing elements of five diverse fungi. *Eukaryotic Cell*.

[44] Fahimeh Charkazi S. S. R., Soltanloo H. (2010). Expression pattern of two sugar transporter genes (SuT4 and SuT5) under salt stress in wheat. *Plant Omics*.

[45] He B., Tu Y., Hu Z. (2018). Genome-wide identification and expression profile analysis of the HOG gene family in Aspergillus oryzae. *World Journal of Microbiology and Biotechnology*.

[46] Han F., Pei H., Hu W., Zhang S., Han L., Ma G. (2016). The feasibility of ultrasonic stimulation on microalgae for efficient lipid accumulation at the end of the logarithmic phase. *Algal Research*.

[47] Elisabeth Truernit J. S., Epple P., Illig J., Sauer N. (1996). The sink-specific and stress-regulated Arabidopsis SPP4 gene: enhanced expression of a gene encoding a monosaccharide transporter by wounding, elicitors, and pathogen challenge. *The Plant Cell*.

