# Supplementary material for: Identification and Expression Analysis of Sugar Transporter Gene Family in Aspergillus oryzae
Source: Int J Genomics. 2020 Nov 7;2020:7146701. doi: 10.1155/2020/7146701 (PMC7666707; doi:10.1155/2020/7146701)
Supplement: Supplementary 1 — Table S1: primer details for genes selected for qRT-PCR analysis in this study. [file 7146701.f1.pdf]

Table S1: Primers used in this study.

Primer	Sequence (5′-3′)	Purpose
AoSUT1-F	GGTCGTCTACATTTGCGAGATA	qRT-PCR of AoSUT1
AoSUT1-R	ACCACCAATCCCAGTGTAATC	qRT-PCR of AoSUT1
AoSUT5-F	GCCCTCCCGTTCTGATTATT	qRT-PCR of AoSUT5
AoSUT5-R	GCGATAAAGGATGACGCTACT	qRT-PCR of AoSUT5
AoSUT12-F	GATGGAACACATGAGCATAGGA	qRT-PCR of AoSUT12
AoSUT12-R	GCAAGCGCCTTAAATGGATTAG	qRT-PCR of AoSUT12
AoSUT23-F	GTTCTACTCGGTTGGTTCCTTTA	qRT-PCR of AoSUT23
AoSUT23-R	AACCATCTTCCAGTCCCATTC	qRT-PCR of AoSUT23
AoSUT35-F	GTCACCTCGATTCCTCATCTAC	qRT-PCR of AoSUT35
AoSUT35-R	CTGAACCACTTCGTCCTCTT	qRT-PCR of AoSUT35
AoSUT69-F	AGAGAGTATCAGGTCTAGGTAT GG	qRT-PCR of AoSUT69
AoSUT69-R	CGGTCGTGATGGGAGAATAAA	qRT-PCR of AoFAD69
18S-F	TTACCCAATCCCGACAC	qRT-PCR of reference gene
18S-R	CCAGACTTGCCCTCCA	qRT-PCR of reference gene
