# Supplementary material for: Identification and Expression Analysis of Sugar Transporter Gene Family in Aspergillus oryzae
Source: Int J Genomics. 2020 Nov 7;2020:7146701. doi: 10.1155/2020/7146701 (PMC7666707; doi:10.1155/2020/7146701)
Supplement: Supplementary 2 — Table S2: the detailed information of the AoSUT proteins in the PPI network. [file 7146701.f2.pdf]

Table S2： The detailed information of the proteins in PPI network

**Source**	**Target**
Serine	AoSUT52
AoSUT97	Shikimate dehydrogenase
AoSUT100	Vacuolar transport chaperone
AoSUT49	Vacuolar transport chaperone
Shikimate dehydrogenase	Uncharacterized protein
AoSUT67	Neutral trehalase
AoSUT97	Uncharacterized protein
Vacuolar transport chaperone	AoSUT109
Vacuolar transport chaperone	AoSUT33
Vacuolar transport chaperone	AoSUT26
AoSUT91	Vacuolar transport chaperone
AoSUT39	AoSUT67
Serine	AoSUT16
Serine	AoSUT38
AoSUT44	AoSUT7
AoSUT71	AoSUT44
AoSUT70	AoSUT22
AoSUT47	AoSUT22
AoSUT50	AoSUT22
AoSUT80	AoSUT6
AoSUT55	AoSUT44
AoSUT44	AoSUT88
AoSUT44	AoSUT5
AoSUT72	AoSUT44
