# Supplementary material for: Identification and Expression Analysis of Sugar Transporter Gene Family in Aspergillus oryzae
Source: Int J Genomics. 2020 Nov 7;2020:7146701. doi: 10.1155/2020/7146701 (PMC7666707; doi:10.1155/2020/7146701)
Supplement: Supplementary 3 — Figure S1: phylogenetic analysis of A. oryzae SUT proteins. An unrooted NJ tree of A. oryzae SUT amino acid sequences using 1000 bootstrap replicates by MEGA X. The eight clades are marked by different colors. [file 7146701.f3.pdf]

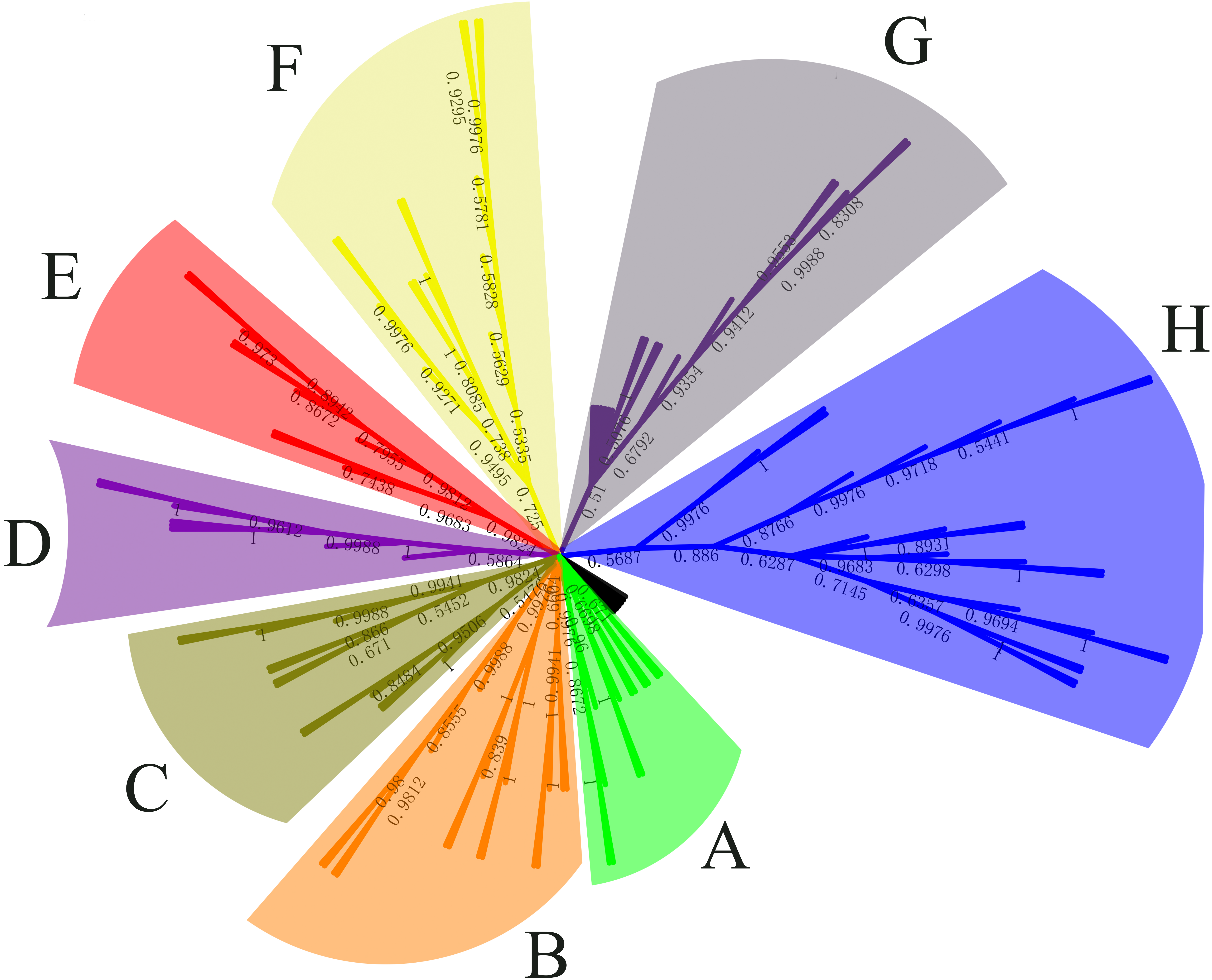

Figure S1: Phylogenetic analysis of *A. oryzae* SUT proteins. An unrooted NJ tree of A. oryzae SUT amino acid sequences using 1000 bootstrap replicates by MEGA X. The eight clades are marked by different colors.
