# Supplementary material for: Identification and Expression Analysis of Sugar Transporter Gene Family in Aspergillus oryzae
Source: Int J Genomics. 2020 Nov 7;2020:7146701. doi: 10.1155/2020/7146701 (PMC7666707; doi:10.1155/2020/7146701)
Supplement: Supplementary 4 — Figure S2: sequence logos for 6 motifs of sugar transporter (SUT) domains using the MEME program. MEME motifs are displayed by stacks of letters at each site. The total height of the stack is the “information content” of that site in the motif in bits. The height of each letter in a stack is the probability of the letter at that site multiplied by the total information content of the stack. Abscissa represents the width of the motif, and ordinate represents the bits of each letter, separately. [file 7146701.f4.pdf]

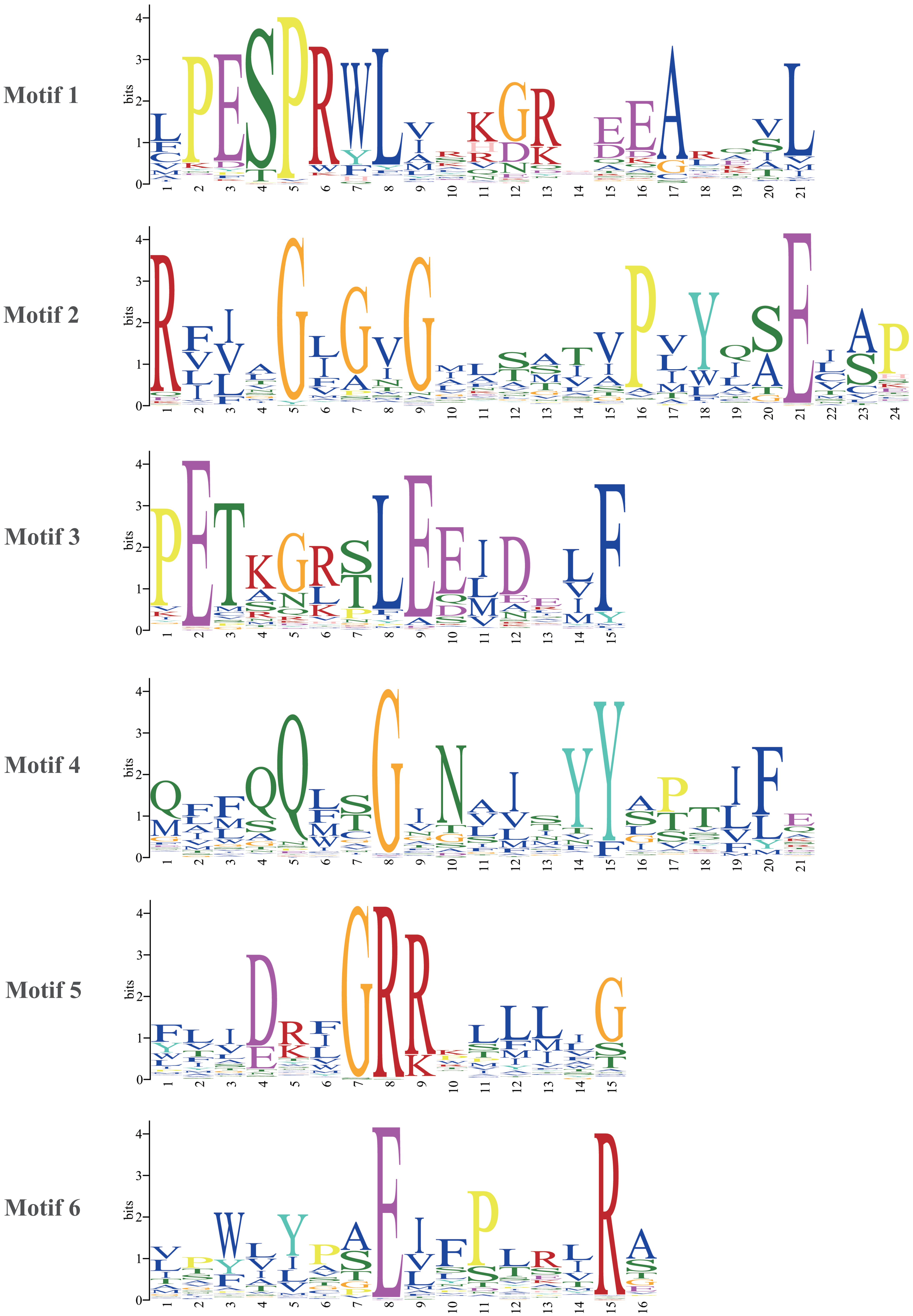

Figure S2: Sequence logos for 6 motifs of sugar transporter (SUT) domains using the MEME program. MEME motifs are displayed by stacks of letters at each site. The total height of the stack is the 'information content' of that site in the motif in bits. The height of each letter in a stack is the probability of the letter at that site multiplied by the total information content of the stack. Abscissa represents the width of the motif and ordinate represents the bits of each letter, separately;
