# Supplementary material for: Identification and Expression Analysis of Sugar Transporter Gene Family in Aspergillus oryzae
Source: Int J Genomics. 2020 Nov 7;2020:7146701. doi: 10.1155/2020/7146701 (PMC7666707; doi:10.1155/2020/7146701)
Supplement: Supplementary 5 — Figure S3: exon-intron structure of 127 sugar transporter genes identified in A. oryzae. Exons are shown as red boxes, and introns are shown as black lines. [file 7146701.f5.pdf]

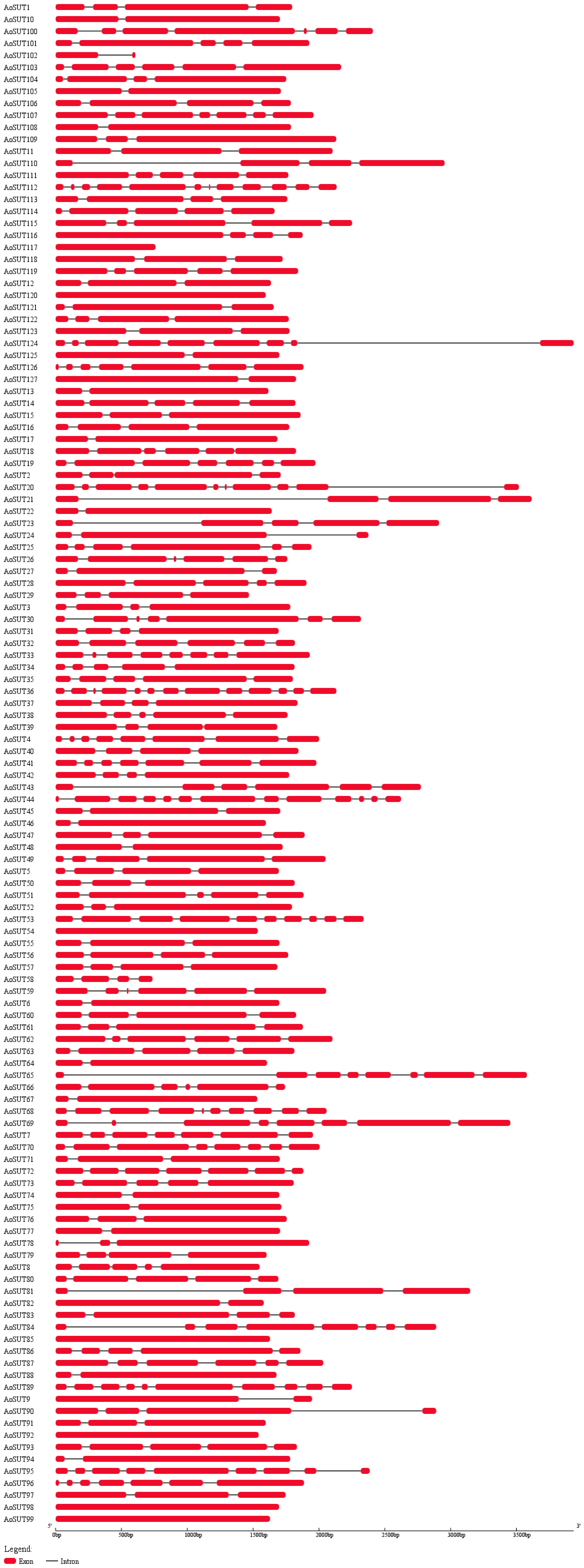


Figure S3: Exon–intron structure of 127 sugar transporter genes identified in *A. oryzae*.
Exons are shown as red boxes, and introns are shown as black lines.
