# Supplementary material for: Identification and Expression Analysis of Sugar Transporter Gene Family in Aspergillus oryzae
Source: Int J Genomics. 2020 Nov 7;2020:7146701. doi: 10.1155/2020/7146701 (PMC7666707; doi:10.1155/2020/7146701)
Supplement: Supplementary 6 — Figure S4: the phylogenetic relationship between A. oryzae, A. niger, and Tuber melanosporum SUT proteins. The maximum likelihood tree was constructed by the MEGA X with 1000 bootstrap replications. Nine clades were distinguished in different colors. [file 7146701.f6.pdf]

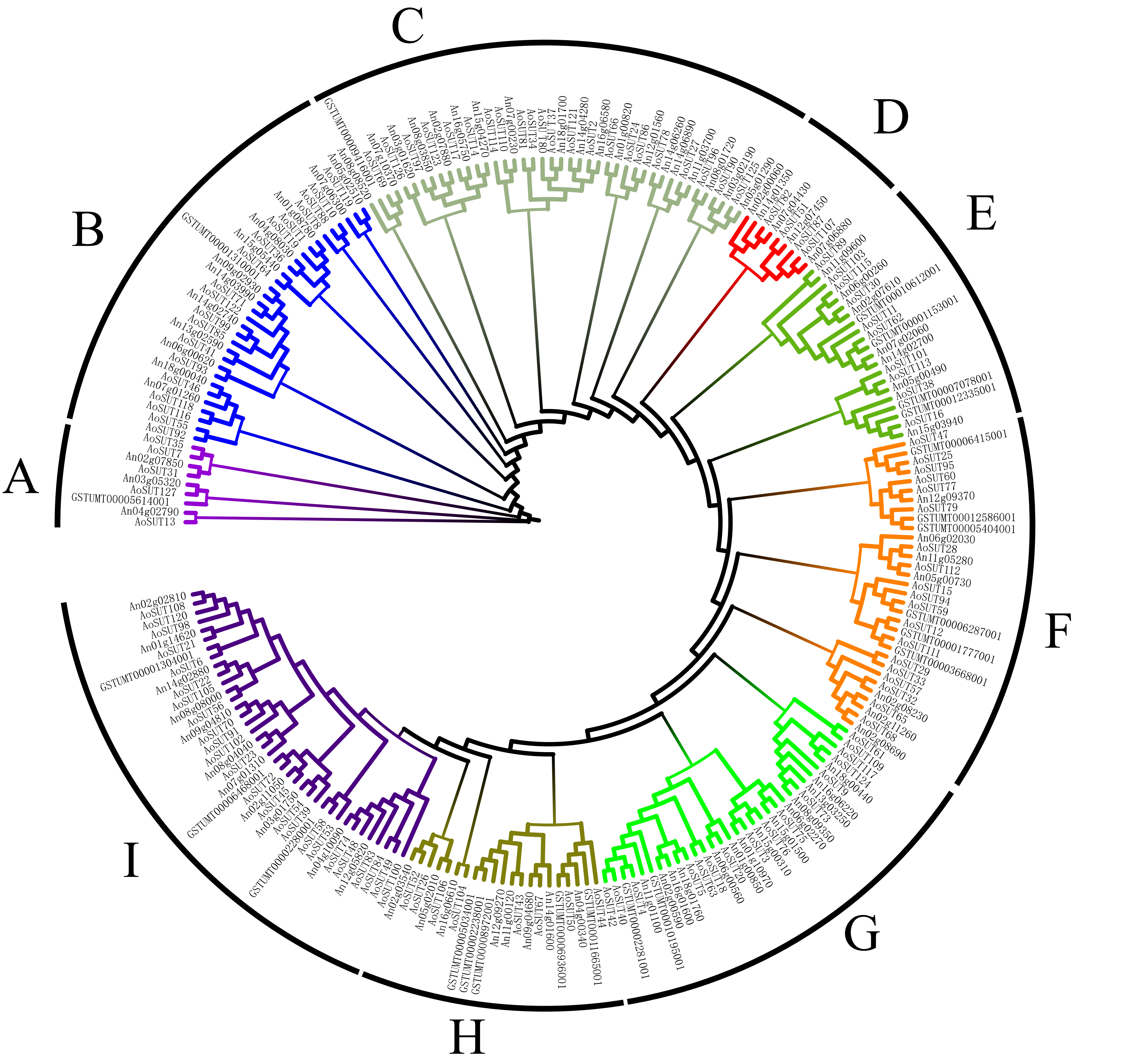

Figure S4: The phylogenetic relationship between *A. oryzae*, *A.niger* and *Tuber melanosporum* SUT proteins. The maximum likelihood tree was constructed by the MEGA X with 1 000 bootstrap replications. Nine clades were distinguished in different colors.
